# Latrine coverage and its utilisation in a rural village of Eastern Nepal: a community-based cross-sectional study

**DOI:** 10.1186/s13104-017-2539-3

**Published:** 2017-06-12

**Authors:** Shyam Sundar Budhathoki, Gambhir Shrestha, Meika Bhattachan, Suman Bahadur Singh, Nilambar Jha, Paras K. Pokharel

**Affiliations:** School of Public Health and Community Medicine, B.P. Koirala Institute of Health Sciences, Dharan, Nepal

**Keywords:** Latrine coverage, Open field defecation, Open defecation free initiative, Sanitation in rural Nepal

## Abstract

**Background:**

A little more than 1/3rd of the rural households in Nepal have improved latrine facility. The government of Nepal is working towards making an open defecation free area all over Nepal. There is no data found in literature searches regarding the status of latrines and its utilisation in Nepal. This study aims to estimate the coverage and utilisation of latrine and its associated factors in a rural community of Nepal.

**Methods:**

We conducted a cross sectional study in March 2015–September 2015 among 625 households in Hattimuda Village, Morang district in Eastern Nepal using semi-structured pre-tested questionnaire with observational checklists.

**Results:**

Out of 623 households, 473 (76.9%) have latrine facilities. There is an increase in latrine coverage in Hattimuda by 37% (38.9% in 2011 to 75.9% in 2016). Majority of the latrines (89.9%) were functional, however 32.3% needs maintenance. The extent of latrine utilisation among those households with a toilet at home was satisfactory (94.3%). Presence of child below 5 years of age at home (OR 2.37, 95% CI 0.05–0.46), functional latrine (OR 27.37, 95% CI 6.84–109.45), frequency of cleaning (OR 3.66, 95% CI 1.09–12.29) and latrine constructed with self-initiation (OR 4.21, 95% CI 1.06–16.66) are factors significantly associated with the utilisation of the latrine.

**Conclusions:**

While the coverage needs to be increased, appropriate interventions to increase the utilisation of latrine needs to be in place so that the village moves closer to open defecation free (ODF) status. As other studies are not found from Nepal, the findings from this study can be used a reference for other rural areas of Nepal.

**Electronic supplementary material:**

The online version of this article (doi:10.1186/s13104-017-2539-3) contains supplementary material, which is available to authorized users.

## Background

Worldwide, the disease burden associated with poor water, sanitation, and hygiene is estimated to account for 4.0% of all deaths and 5.7% of the total disease burden in disability-adjusted life year. These deaths occur principally due to diarrhoeal diseases, schistosomiasis, trachoma, ascariasis, trichuriasis, and hookworm infection [1, 2]. Diarrhoea accounts for the largest share of sanitation-related morbidity and mortality, causing an estimated 1.4 million deaths annually [3].

More than 2.5 billion people in the world are still lacking access to improved sanitation and more than half of these people reside in low and middle-income countries. Globally, about 15% of the population still practice open defecation [4]. An estimated 1.1 billion people practice open defecation, exposing themselves and the community to major health risks [4, 5].

Having a latrine at home is found to be a protective factor for communicable diseases [6, 7]. About 52% of the population practice proper utilisation of latrine in low income countries [8, 9]. Improper utilisation of sanitary facilities leads to the contamination of the water sources. Proper utilisation of sanitary facilities helps to interrupt diseases transmitted through the faecal-oral route [10].

Efforts to increase access to safe water and improved latrine thus help to promote the proper utilisation of sanitary facilities [11]. An example of such efforts can be seen in neighbouring- India “*Take Poo to the Loo”* campaign, which seeks to drive nationwide change by influencing public opinion to call for an end to open defecation in India [12].

### Sanitation situation in Nepal

In Nepal, improper sanitation is a major cause of diarrhoea [13]. According to Nepal Demographic Health Survey 2011 report, 38% of households have improved toilet facility, 53% urban and 36% rural households. The majority of households, 43% (48% rural and 11% urban) use non-improved latrine facilities. About 36% of the households still use a bush or open field for defecation [14].

Presence of improved latrine in the household is associated with safe disposal practices for child faeces [15]. Majority of the population in rural areas practice open defecation. Improvement in the sanitary habit of the people is essential for overall improvement in health status of the people. Both coverage and the use of latrine are essential for the health benefit of the community associated with improved sanitation [16].

### Sanitation initiatives in Nepal

Sanitation improvement programs in combination with water supply projects began in 1990s in Nepal. The programs expanded over the years with different non-governmental agencies supporting the sanitation improvement activities to increase toilet coverage. Community Led Total Sanitation (CLTS), which began in 2003 and School Led Total Sanitation (SLTS) in 2006 are approaches that emphasized on creating open defecation free (ODF) communities in Nepal. This ODF approach became popular over time with Nepal declaring its first ODF village (Ghachok) in 2007 and district (Kaski) in 2011 [17]. The Sanitation and Hygiene masterplan 2011 has highlighted ODF approach as a priority sanitation initiative [17]. Government of Nepal is committed to making Nepal open defecation free (ODF) by 2017. This initiative targets to bring down the faecal-oral transmission of diseases in Nepal [18]. In order to reach the country wide ODF status, the sanitation and hygiene master plan 2011 of Nepal aims for all households in Nepal to have access to toilet by 2017 [17].

There are very few studies found in Nepal that report on the status and utilisation of latrines, especially in Eastern Nepal. Therefore, we conducted this cross sectional study to assess the utilisation of latrine in a rural community. The study aims to estimate the coverage and utilisation of latrine in Hattimuda village and to examine the factors associated with the utilisation of latrines.

## Methods

### Study setting and design

We conducted a community-based cross-sectional study in Hattimuda village, Morang district, Eastern Nepal from March 2015 to September 2015. We chose Hattimuda village purposively, as it is the nearest village to the Comprehensive Health Service Area, a community hospital in the rural field practice area of School of Public Health and Community Medicine, B P Koirala Institute of Health Sciences, Dharan, Nepal. Community medicine teaching and learning activities for undergraduate and postgraduate medical students takes place in the rural field practice area covered by the hospital. The local government is working towards declaring Hattimuda village as an open defecation free village. Therefore, the findings of the study could also provide evidence to the government to assist Hattimuda reach the open defecation status. We chose the village so that we can use the findings from the study to design community led activities for hygiene promotion. The purpose was also to be able to longitudinally follow-up the interventions and measure the impact of our community based activities. We conducted this study prior to starting our community based teaching and learning activities in Hattimuda, so this study can also provide a baseline information on latrine utilisation for Hattimuda village.

#### Sanitation situation in Hattimuda

Hattimuda village occupies the total area of 13.56 km^2^. According to National Housing and Population Census 2011, the population of the village is 10,250 of which 5141 are males and 5109 are females. In 2011, the total number of households in the area is 2206 of which only 38.9% have the toilet facility [16]. Following the national campaign, Hattimuda has also the target to attain open defecation free status by 2017. The activities by local government for sanitation are: formation of sanitation sub-committees in the villages, fortnightly door-to-door campaigns to educate the households regarding latrine construction and its use, disseminate sanitary awareness message through community groups (school teachers’ group, mothers’ group, students’ network and health volunteers’ groups), and material support to the socio-economically marginalized households to construct toilets [19].

### Sample size

In National Population and Housing Census 2011, the percentage of households with toilet facility in Hattimuda village was 38.9% [16]. By using a single population proportion formula considering the coverage of latrine as 38.9 at 95% confidence interval and relative precision of 10%, the required sample size was 625 households.

### Sampling technique

Out of the 9 wards in Hattimuda village, 5 wards were chosen randomly using lottery method (Additional file 1). We selected every second house for an interview until sample size was reached proportionate to population size in the respective wards.

### Data collection

We collected the data by face-to-face interviews using pretested semi-structured questionnaires, which included socio-demographic characteristics of the household, status of the latrine, its utilisation, sanitary behaviour and an observation checklist for latrine. Latrine utilisation was assessed by asking if all family members above 5 years use latrines or not. We assumed that people above 5 years should be able to use the latrine, hence 5 years cut off value was taken. One family member above 16 years of age, preferably head of the household was included in the study. Family who have rented the house; and living in Hattimuda for less than 12 months and households with latrine under construction in the study period were excluded from the study.

### Operational definitions

Functional latrine was defined as the latrine with walls over 1.5 ms, some type of closure over the entry, unbroken and unblocked pan and a functional pan-pipe-pit connection [20].

Latrine utilisation was defined as the use of the latrine by all the family members (above 5 years) in the households, that own private latrines.

Economic status of the family was categorized into below poverty line, which is per capita income less than 1.25 dollars per day per person and above poverty line, which is per capita income more than or equals to 1.25 dollars per day per person (1 US dollar ≈ 100 Nepalese rupees) [21].

Occupation was classified according to Nepal Standard Occupations Classification—99 [22].

Houses were categorized into 3 types: Pucca with the floor, walls and roof all cemented, semi-pucca in which any one of the floors, walls, or roof is cemented and kuccha where none of the floor, walls and roof is cemented.

### Statistical analysis

We entered the data into MS-Excel 2007 software followed by the Statistical Package for Social Sciences (version 17) for data analysis. Dependent variable was the utilisation of latrine among those with age above 5 years. Independent variables in the study included, the socio-demographic variables and the latrine related variables (age of the head of the household, education, family size, family type, house type, child under 5 years of age, economic status, distance of latrine from the kitchen, type of latrine, years of latrine construction, frequency of latrine cleaning, functionality of latrine, need of maintenance, reason for latrine construction, and the frequency of cleaning latrine).

The coverage and utilisation of latrine was expressed using descriptive data analysis. The Chi square test was performed to determine the association of utilisation of latrine with the independent variables. A *p* value of <0.05 was considered as the cut-off point for statistical significance. Variables with p value <0.20 were further analyzed with multiple logistic regression to find out the strength of association between the variables.

## Results

We approached 625 households were approached for the study. Two households did not agree to participate and 623 households were included in this study. The overall response rate was 99.7%.

### Characteristics of the households

Most of the respondents (57.6%) were female. The mean age of the respondents was 36.1 ± 40.3 years and ranged from 16 to 84 years. Table 1 displays the socio-demographic characteristics of the 623 households. Among 623 households, 473 (75.9%) had latrine at home. Those who did not have a latrine, the informants reported that all members of the household practice open defecation.

**Table 1:** Characteristics of the households in Hattimuda village (n = 623)

Socio-demographic characteristics	Categories	Number (n)	Percentage (%)
Gender of the informants	Male	264	42.4
	Female	359	57.6
Age of informant (in years)	<30	215	34.5
	30–49	287	46.1
	>50	121	19.4
Age of the head of household (in years)	<30	45	7.2
	30–49	356	57.1
	>50	222	35.6
Religion	Hindu	476	76.4
	Others	147	23.6
Family size	≤5	339	54.4
	>5	284	45.6
Family type	Nuclear	407	65.3
	Joint	216	34.7
Under 5 year child in family	Present	256	41.1
	Absent	367	58.9
House type	Kuccha	288	46.2
	Semi-pucca	231	37.1
	Pucca	104	16.7
Education of the head of the household	Illiterate	322	51.7
	Literate	301	48.3
Economic status	Below poverty line	476	76.4
	Above poverty line	147	23.6
Presence of latrine at home	Present	473	75.9
	Absent	150	24.1

### Latrine utilisation and reasons for latrine construction

Latrine utilized by all members above 5 years was 94.3%. Self-initiation was the reason for the construction of latrine for 67.4% of the households while 16.5% credited advice from the health workers (Table 2).

**Table 2:** Latrine utilisation, reasons for construction and latrine characteristics in the households of Hattimuda village (n = 473)

Characteristics	Categories	Number (n)	Percentage (%)
Utilisation of latrine	Yes	446	94.3
	No	27	5.7
Reasons for latrine construction	Advice from health workers	78	16.5
	Self-initiation	319	67.4
	Peer pressure	49	10.4
	Imposition from others	27	5.7
Type of latrine	Direct pit	32	6.8
	Pour flush	441	93.2
Distance of latrine from kitchen (meter)	<6	358	75.7
	6–10	90	19.0
	>10	25	5.3
Years since latrine constructed	<2	225	47.6
	≥2	248	52.4
Latrine cleaning	Always	309	65.3
	Often	101	21.4
	Rarely	63	13.3
Assistance from government/NGO to build latrine	Yes	237	50.1
	No	236	49.9
Wall height over 1.5 m	Yes	423	89.4
	No	50	10.6
Closure for privacy	Yes	434	91.8
	No	39	8.2
Functional	Yes	425	89.9
	No	48	10.1
Need for maintenance	Yes	153	32.3
	No	320	67.6

### Characteristics of the latrines

Most of the latrines (93.2%) were pour flush type and the rest were of direct pit type. About 75.7% of latrines were at the distance of less than 6 m away from the kitchen. Among those households with latrines, 225 (47.6%) latrines were constructed within last 2-year period. About 65.3% households with latrines, always clean their latrine after defecation. Half (50.1%) of the respondents reported that Government or Non-Governmental Organization (NGO) helped them in some ways to construct their latrines. There were 89.4% of the latrines with wall over 1.5 ms and closure for privacy, which was also functional among 473 latrines. We found that there were 32.3% latrines for need of maintenance (Table 2).

### Factors associated with the use of latrine in Hattimuda

Utilisation of latrines was less (OR 0.26, 95% CI 0.11–0.62) in the households with child below 5 years. Latrines constructed within 2 years (OR 0.18, 95% CI 0.07–0.51), latrines needing maintenance (OR 0.21, 95% CI 0.09–0.49) and latrines built with any assistance from government or NGO (OR 0.33, 95% CI 0.13–0.80) were less likely to be utilised. Functional latrines (OR 18.81, 95% CI 8.06–43.92), latrine height more than 1.5 m (OR 12.26, 95% CI 5.35–28.07), latrines with closure for privacy (OR 21.97, 95% CI 9.26–52.11) and latrines cleaned always (OR 3.45, 95% CI 1.54–7.73) were more likely utilised.

The age of the head of the household, family size, family type, socioeconomic status, literacy status of the head of the household, type of latrine and distance of the latrine from the kitchen was not associated with the utilisation of the latrine in Hattimuda.

When the variables whose p value was taken as <0.20 were analysed on multivariate logistic regression, presence of child above 5 years, use of functional latrine, frequent cleaning of latrine and self-initiation of latrine construction were found to be significant factors for latrine utilisation.

The households having children less than 5 years were less likely to use latrine than those without child (AOR 0.15, 95% CI 0.05–0.46). The households with functional latrines were 27.37 times more likely to utilise latrine than those without a functional latrine (AOR 27.37, 95% CI 6.85–109.45). People from households who always clean latrine were 3.66 times (AOR 3.66, 95% CI 1.09–12.29) more likely to utilise latrine than those who rarely clean latrine. The people from households who built latrine on self-initiation were 4.22 times more likely to use the latrine (AOR 4.21, 95% CI 1.06–16.66) (Table 3).

**Table 3:** Association of latrine utilisation with socio-demographic variables and latrine characteristics—multiple logistic regression analyses (n = 473)

Characteristics	Number (%)	Total	Unadjusted OR (95% CI)	p value	AOR (95% CI)	p value
Socio-demographic variables						
Age of head of the household <50 years	280 (94.6)	293	1.15 (0.52–2.55)	0.714	–	–
Family size ≤5 members	240 (94.9)	253	1.25 (0.57–2.73)	0.567	–	–
Joint family	171 (95.0)	180	0.08 (0.35–1.83)	0.603	–	–
Presence of under 5 children	173 (90.1)	192	0.26 (0.11–0.62)	<0.001*	0.15 (0.05–0.46)	0.001*
Semi pucca housing	276 (95.8)	288	0.49 (0.22–1.07)	0.071	1.33 (0.444–4.02)	0.605
Illiterate head of the household	212 (93.4)	227	0.90 (0.43–1.89)	0.418	–	–
Economic status below poverty line	330 (93.8)	352	0.64 (0.23–1.74)	0.386	–	–
Characteristics of latrine						
Flush type latrine	421 (95.5)	441	0.16 (0.06–0.43)	<0.001*	2.37 (0.56–9.99)	0.240
Duration of latrine construction ≥2 years	243 (98.0)	248	0.18 (0.07–051)	<0.001*	3.02 (0.84–10.91)	0.090
Distance of latrine from kitchen < 6 m	337 (94.8)	358	0.88 (0.34–2.24)	0.794	–	–
Functional latrine	414 (92.8)	425	18.81 (8.06–43.92)	<0.001*	27.37 (6.84–109.45)	<0.001*
Need for maintenance	135 (88.2)	153	0.21 (0.09–0.49)	<0.001*	1.64 (0.46–5.83)	0.439
Height of latrine >1.5 m	410 (96.9)	423	12.26 (5.35–28.07)	<0.001*	–^a^	–
Closure for privacy	422 (97.2)	434	21.97 (9.26–52.11)	<0.001*	–^a^	–
Frequency of latrine cleaning as always	299 (96.8)	309	3.45 (1.54–7.73)	<0.05*	3.66 (1.09–12.29)	0.036*
Help from Government/NGO	217 (91.6)	237	0.33 (0.13–0.80)	<0.05*	0.72 (0.22–2.87)	0.727
Self initiation of latrine construction	304 (95.3)	319	1.71 (0.78–3.75)	0.175	4.21 (1.06–16.66)	0.040*

^a^Variables not entered in multiple logistic regression since these variables represents functionality of latrine

* Statistically significant

## Discussion

This study assessed the latrine coverage and its utilisation in a village of eastern Nepal. While the government of Nepal is committed to improve sanitation throughout the country, one priority campaign is improving latrine coverage towards attaining open defecation free areas all over the country by 2017 [17]. This community based cross-sectional study provides evidence that while the coverage is improving utilisation of the constructed latrines may also need to improve through appropriate community based approaches. Among the 623 selected households, 75.9% households possessed latrine. While 24.1% households who did not possess latrine, practiced open field defecation, and 5.7% of those having latrine at home still practiced open field defecation. A study from Bhopal, India also report similar proportion of people practicing open field defecation [23]. The latrine coverage of Hattimuda (75.9%) from this study is higher than the national data (72.1%), latrine coverage of Morang district (32.6% as of 2015 [24]. According to the National Census data of 2011, the latrine coverage of Hattimuda was 38.9% and the National Coverage was 61.9% [16]. Aiming for attaining the open defecation free status by 2017 [19], the coverage of latrine in Hattimuda has increased. The sanitary awareness and support campaigns implemented by the local government itself and the non-governmental organizations in the recent years to make the village open defecation free area are ongoing in Hattimuda [18, 25]. The impact of these individual programs on sanitation situation of the village is not available. As we purposively selected Hattimuda village closer to the community hospital, there may seem a space to question the generalisability of the results to other villages in Nepal. However, the socio-economic status, population density, living characteristics are similar to the other nearby villages [16]. However, we did not find other literatures regarding utilisation of the latrine; we believe that this study could still be used as evidence from rural Nepal. As Hattimuda village is nearer to the community hospital, the people may be more aware about the health consequences of not having a latrine and poor sanitation. The village nearer to the hospitals are expected have more access to health education and awareness campaigns. Assuming this, if the village closer to the hospitals are not fully utilising the latrines, means that the other villages will need more attention. However, we selected the village, also with the purpose of conducting follow up health promotion activities through the hospital.

Around 95% of household utilised the latrine in this study while it was reported to be 84% in rural Ethiopia [9], 38% in rural India [20] and 8.8% in a study from Ethiopia [9]. A study from Uganda, reports of children not utilising the latrine despite its availability [26]. This could be due to possible differences in toilet behaviour practices among children from our settings.

In this study, there was no association between the age of the head of the household and latrine utilisation. This finding is in accordance with the studies done in Tanzania [27]. We found no significant association with the socioeconomic status of the people of Hattimuda and latrine utilisation. Nevertheless, some studies have shown a significant relationship between income and latrine utilisation [28].

The households with a child less than 5 years were associated with under utilisation of latrine in the present study, which is similar to the study done in Ethiopia [9]. The reason may be children less than 5 years cannot use the latrine properly so they practice open field defecation and those members accompanying these children could have practiced open field defecation.

Utilisation of the latrine was higher in households who took their own initiative to construct their latrine. This is accordance with findings from Ethiopia [8]. As people learn to accept, adopt and utilise latrine facilities easily by perceiving on their own and observing model latrine facilities than mere advice and enforcement. In contrast, utilisation of latrine was lower among households who constructed latrines with subsidy from Government/Non-Government Organisation (NGO). Possible reason for lesser frequency of latrine use could be the low sense of ownership of the latrine in the household where latrine was not constructed based on the felt need of the family. The use was also lower in the households that had no financial contribution to their latrine construction.

Our study did not find an association between the duration of owning the latrine and its utilisation. This was found significant in the unadjusted model, which was not significant later in the adjusted model. Studies from Ethiopia [29] and India [20] however found an association. This may be due to the behavioural patterns that may be different from the countries that could influence latrine utilisation in Hattimuda, which may require further exploration.

In our study, we found an association of latrine utilisation with the type of latrine, which is similar to the study done in Tanzania [27]. Flush latrines are more likely to be utilised than the pit latrines. The probable reason may be fear of odour and nuisance created by houseflies linked with traditional pit latrines.

The utilisation of latrines depended on their functionality and need of maintenance in Hattimuda as also reported in Ethiopia [8]. Non-functional latrine gives various problems such as leakage, privacy issues, lack of comfort that may hinder its use. Similarly, presence of door and height of latrine above 1.5 m was positively associated with latrine utilisation. These findings are similar to the study done in India and Ethiopia [20, 29].

The frequency of cleaning of the latrine was associated with the utilisation of latrine in this study. This is analogous to the study done in Ethiopia [9]. Households who clean their latrine more frequently were more likely to utilise their latrine than those who clean their latrine less frequently. This is consistent with other studies which concluded that foul odour is a determinant for latrine non-utilisation [29, 30].

## Limitations of the study

As this a cross-sectional study, we could only find the association between the factors and the latrine utilisation, the causality could not be established. The purposive selection of the study site closer to the hospital could have influenced the responses by the respondents.

## Conclusions

Latrine coverage in the Hattimuda village is improving. Utilisation of the latrine was better in households, when the latrine was functional, cleaned regularly, built by self-initiation, and with the presence of a child under 5 years. The factors identified may be useful for practitioners working for open defecation free initiative in Nepal to promote latrine utilisation. Interventions focusing on motivating the building of latrines at households could improve the utilisation. There is a need to identify locally appropriate interventions to improve latrine coverage and utilisation in the local context.

## Additional file

**Additional file 1.** Table showing sample size in randomly selected wards in Hattimuda VDC, Morang District, Nepal.

## Abbreviations

AOR: adjusted odds ratio
CI: confidence interval
NGO: Non-Government Organisation
ODF: open defecation free
OR: odds ratio
