# Supplementary material for: Latrine coverage and its utilisation in a rural village of Eastern Nepal: a community-based cross-sectional study
Source: BMC Res Notes. 2017 Jun 12;10:209. doi: 10.1186/s13104-017-2539-3 (PMC5469064; doi:10.1186/s13104-017-2539-3)
Supplement: Supplementary file 1 — Additional file 1. Table showing sample size in randomly selected wards in Hattimuda VDC, Morang District, Nepal. [file 13104_2017_2539_MOESM1_ESM.docx]

| **Ward** | **Household^*^** | **Randomly selected** | **Sample size in selected wards** |
| --- | --- | --- | --- |
| 1 | 208 | **Yes** | 95 |
| 2 | 101 | No | 0 |
| 3 | 157 | **Yes** | 72 |
| 4 | 168 | **Yes** | 77 |
| 5 | 147 | No | 0 |
| 6 | 148 | No | 0 |
| 7 | 354 | **Yes** | 161 |
| 8 | 438 | No | 0 |
| 9 | 485 | **Yes** | 221 |
| **Total** | **2206** |  | **625** |
| **Total** **households in selected wards=1372** | | | |

Additional file 1: Table showing sample size in randomly selected wards in Hattimuda VDC, Morang District, Nepal

| ^*^Household number according to National Population Census 2011, Morang, Central Bureau of Statistics. |
| --- |
